# Evaluating the Efficacy of a Mobile Phone App in Enhancing Menopause Knowledge and Shared Decision-Making: Protocol for a Randomized Controlled Trial

**DOI:** 10.2196/76536

**Published:** 2025-10-08

**Authors:** Jana Karam, Maggie M Paul, Chrisandra Shufelt, Prajna Ravikumar, Amy M Fratianni, Sey Oloyede, Erin M Pagel, Mary S Hedges, Ekta Kapoor, Juliana M Kling, Kristin Cole, Rajeev Chaudhry, Stephanie S Faubion

**Affiliations:** 1 Mayo Clinic in Florida Jacksonville, FL United States; 2 Women's Health Department Mayo Clinic Rochester, MN United States; 3 Robert D and Patricia E Kern Center for the Science of Health Care Delivery Mayo Clinic in Arizona Phoenix, AZ United States; 4 Division of Community Internal Medicine Mayo Clinic in Florida Jacksonville, FL United States; 5 Division of Endocrinology, Diabetes, Metabolism, and Nutrition Mayo Clinic Rochester, MN United States; 6 Division of Women’s Health Internal Medicine Mayo Clinic in Arizona Scottsdale, AZ United States; 7 Department of Quantitative Health Sciences Mayo Clinic Rochester, MN United States; 8 Division of Community Internal Medicine Mayo Clinic Rochester, MN United States

**Keywords:** menopause, mobile health, mHealth, randomized controlled trial, RCT, digital health, mobile app

## Abstract

**Background:**

Menopause symptoms are common but often inadequately addressed by primary care clinicians due to limited time for discussions and resources. Mobile health apps can play a crucial role in symptom identification and management; yet, many existing menopause-focused apps lack evidence-based content and medical expertise.

**Objective:**

The aim of this study is to describe the protocol study design and methodology of a randomized controlled trial to evaluate the effectiveness of the emmii mobile app for improving menopause-related knowledge and shared decision-making compared to a traditional menopause education pamphlet.

**Methods:**

This randomized controlled trial will recruit women aged 45-55 years with upcoming primary care appointments at Mayo Clinic within 3 weeks of the date of initial outreach. Eligible patients must be English-speaking, able to provide informed consent, and report a Menopause Rating Scale score ≥5, which indicates that they are experiencing significant menopause-related symptoms. Patients will be randomized to have access to either the emmii app (intervention, n=200) or an evidence-based menopause education pamphlet (control, n=200). The emmii app is developed with direct input from primary care clinicians certified by The Menopause Society and offers symptom tracking, personalized treatment recommendations based on a protocol, and a discussion guide to support communication between patients and their primary care clinicians. Outcomes will include a postappointment survey sent to the patients and their primary care clinicians within 1-3 weeks of the appointment, and assessment of patient knowledge, clinical treatment plans, and both the patient and clinician experience. The study will also compare prescribing rates of hormonal and nonhormonal therapies for menopause symptoms between the emmii intervention and control groups to assess for influence on treatment patterns. Data will be analyzed using descriptive statistics, including chi-square tests, Wilcoxon rank sum tests, and multivariable modeling.

**Results:**

Data collection is scheduled to begin in April 2025.

**Conclusions:**

This protocol outlines the design and methodology of a randomized controlled trial that aims to assess the impact of the emmii app in facilitating menopause care through primary care clinician–patient communication and shared decision-making.

**Trial Registration:**

ClinicalTrials.gov NCT06919887; https://clinicaltrials.gov/ct2/show/NCT06919887

**International Registered Report Identifier (IRRID):**

PRR1-10.2196/76536

## Introduction

### Background

Menopause is defined as the absence of menses for 12 consecutive months or the surgical removal of both ovaries prior to natural menopause [1]. The average age of menopause is 52 years, with natural menopause occurring between the ages of 45 years and 56 years in 90% of the women [1,2]. However, menopause can also occur prematurely (<40 years) in approximately 3% of the women or early (40-44 years) in 6% of the women [3]. Perimenopause, occurring 2-7 years before the final menstrual period through the first year after menopause, is characterized by irregular menstrual cycles [4]. Common symptoms of perimenopause include vasomotor symptoms, mood changes, as well as genitourinary symptoms [5,6]. During perimenopause and menopause, 50%-75% of women experience vasomotor symptoms, 45%-68% experience psychological symptoms, and more than 50% develop genitourinary symptoms [7,8].

Menopause hormone therapy is the first-line treatment for bothersome vasomotor and genitourinary symptoms in symptomatic women without contraindications at and around the time of menopause and may also alleviate menopause-related sleep and psychological symptoms [7-9]. For women with contraindications to hormone therapy or a personal preference not to use it, nonhormonal treatments include selective serotonin reuptake inhibitors, serotonin-norepinephrine reuptake inhibitors, gabapentin, oxybutynin, and fezolinetant, which have shown efficacy for the treatment of vasomotor symptoms [10-13]. Lifestyle interventions such as cognitive behavioral therapy, yoga, and sleep hygiene are also recommended for symptom management [10]. However, these treatments may have limited effectiveness, potential side effects, and require access to trained professionals. As a result, substantial gaps remain in menopause care, leaving many symptomatic women without effective treatment [14]. Barriers also exist in clinical settings, and women may hesitate to report menopause symptoms due to prior negative experiences, discomfort discussing menopause, or the perception that primary care is not the appropriate setting to address such concerns [15,16]. Additionally, clinicians often lack the training or time to routinely screen for and manage menopause-related symptoms [17,18].

With the widespread use of smartphones, mobile health (mHealth) apps have emerged as useful tools for health care management. These apps offer a range of functions—from educational resources and symptom tracking to medical record access and therapeutic guidance. It is estimated that there is at least one app for every medical condition [19,20]. Research suggests that menstrual health and cognitive behavioral therapy apps can enhance physical and psychological well-being [21,22]. A small but growing number of mHealth apps focus specifically on menopause, addressing patient education, symptom tracking, and therapeutic guidance [20]. A longitudinal study of 1900 women using the “Health & Her” app found that those who engaged more frequently with the app’s features such as symptom tracking, period logging, and using in-app activities (eg, reminders to drink water, pelvic floor exercises) experienced significantly greater reductions in menopause symptoms over a 2-month period [23]. A recent randomized controlled trial evaluated the efficacy of Caria, an mHealth app for women experiencing menopause symptoms, which features an artificial intelligence chatbot-assisted symptom assessment and a 6-week self-guided program that includes daily cognitive behavioral therapy lessons, mindfulness-based exercises, physical activity video sessions, and community-based peer support [24]. After 6 weeks, patients randomized to the app had significantly reduced distress related to hot flashes compared to controls. However, the app did not directly facilitate engagement with clinicians or support shared decision-making about management.

Despite their potential benefits, most available menopause mHealth apps do not explicitly support shared decision-making or provide structured, evidence-based education tailored to women’s individual needs. A 2019 systematic review of 22 mHealth menopause apps found that only 27.3% of the apps involved medical professionals in their development, and only 22.7% used evidence-based information, raising concerns about the quality and reliability of these tools [20]. Key gaps include menopause knowledge deficits, lack of guidance on treatment options, limited shared decision-making support, and challenges in symptom self-management. As a result, many women remain underinformed about available therapies or uncertain about how to engage with clinicians regarding their symptoms and preferences.

To address these gaps, the Mayo Clinic Center for Women’s Health, in collaboration with a digital health company, BettrHealth, codeveloped a patient-centered mobile app called emmii for menopause symptom identification and management. The app was designed to provide evidence-based menopause education, individualized symptom assessment, treatment guidance, and a printable discussion guide to facilitate shared decision-making in the primary care setting.

### Objectives

This paper describes the protocol, design, and methodology of a randomized controlled trial, which will evaluate the effectiveness of the emmii mHealth app (emmii) for improving menopause-related knowledge and shared decision-making compared to a traditional menopause education pamphlet. 

## Methods

### App Development

The emmii app was codeveloped through collaboration between the Mayo Clinic Center for Women’s Health and BettrHealth, a digital health company, to offer comprehensive menopause education, evidence-based treatment options, and a personalized discussion guide for patients. It was developed with direct input from health care professionals who are certified by The Menopause Society. The initial app content was informed by clinical guidelines and menopause literature and a review of existing menopause-focused mobile apps, which often lack evidence-based content and do not provide individualized support. To address these limitations, the development process focused on accuracy, personalization, and usability. Content areas were identified and refined based on input from menopause experts and included symptom tracking, treatment education, and shared decision-making support. The app’s educational and treatment content was developed using evidence-based guidelines on menopause symptom management from The Menopause Society, Endocrine Society, and American College of Obstetricians and Gynecologists [9,10,25,26]. The app presents treatment recommendations for both hormone and nonhormone options, including hormone therapy, serotonin reuptake inhibitors/serotonin-norepinephrine reuptake inhibitors, gabapentin, oxybutynin, and fezolinetant, as well as lifestyle interventions [10-13]. These recommendations were created by menopause experts to ensure clinical accuracy. The personalized treatment guidance is based on user-reported symptoms and medical history to support individualized care and shared decision-making.

**Figure 1:**
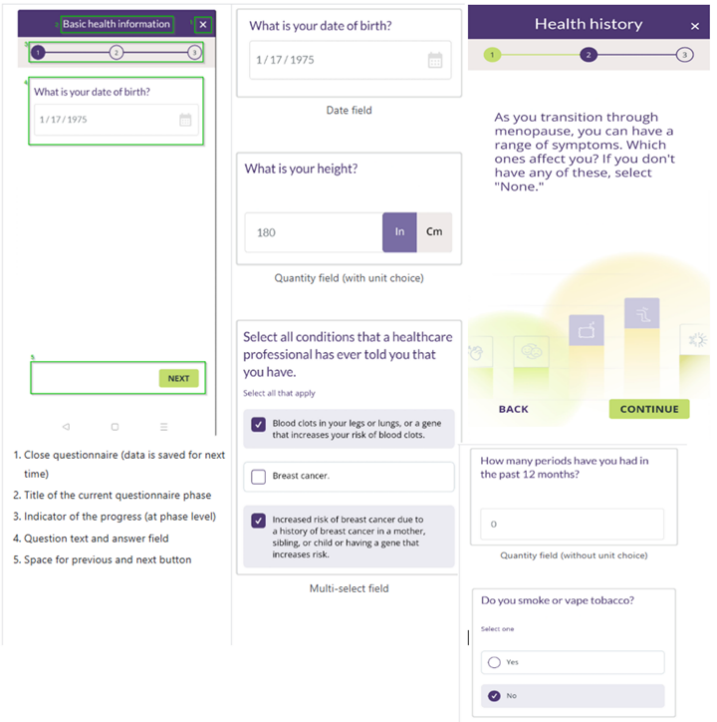
Screenshot of the basic health information and medical history in the emmii app.

**Figure 2:**
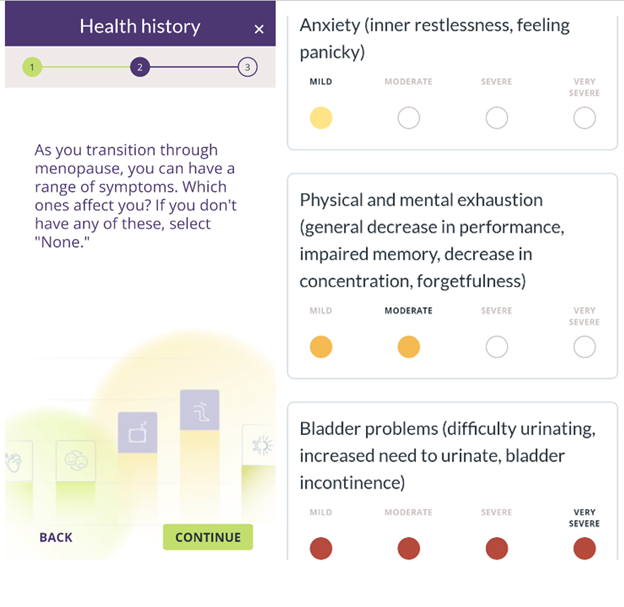
Screenshot of the symptom checker in the emmii app.

**Figure 3:**
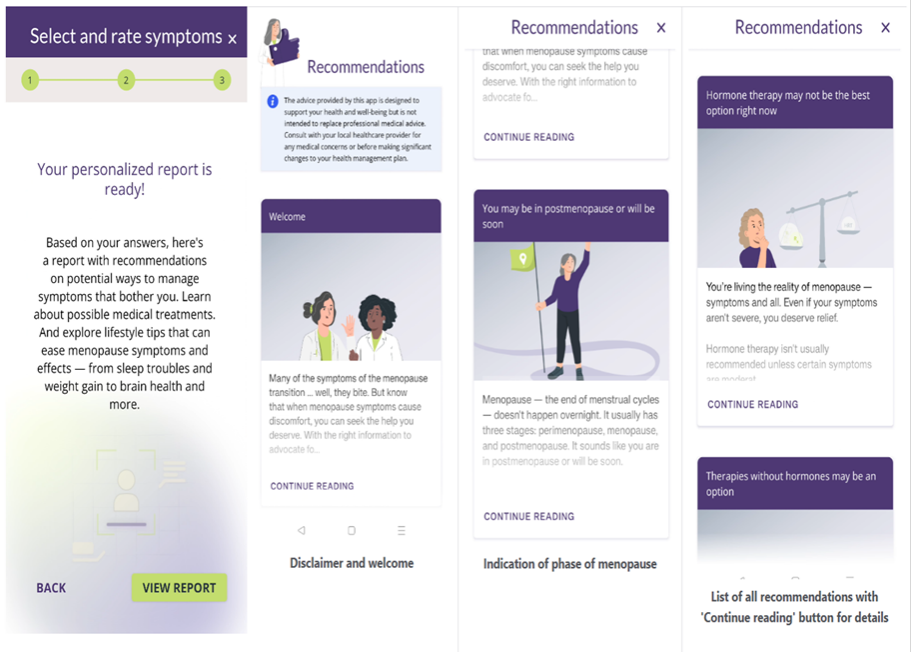
Screenshot of the personalized recommendations generated based on user input in the emmii app.

**Figure 4:**
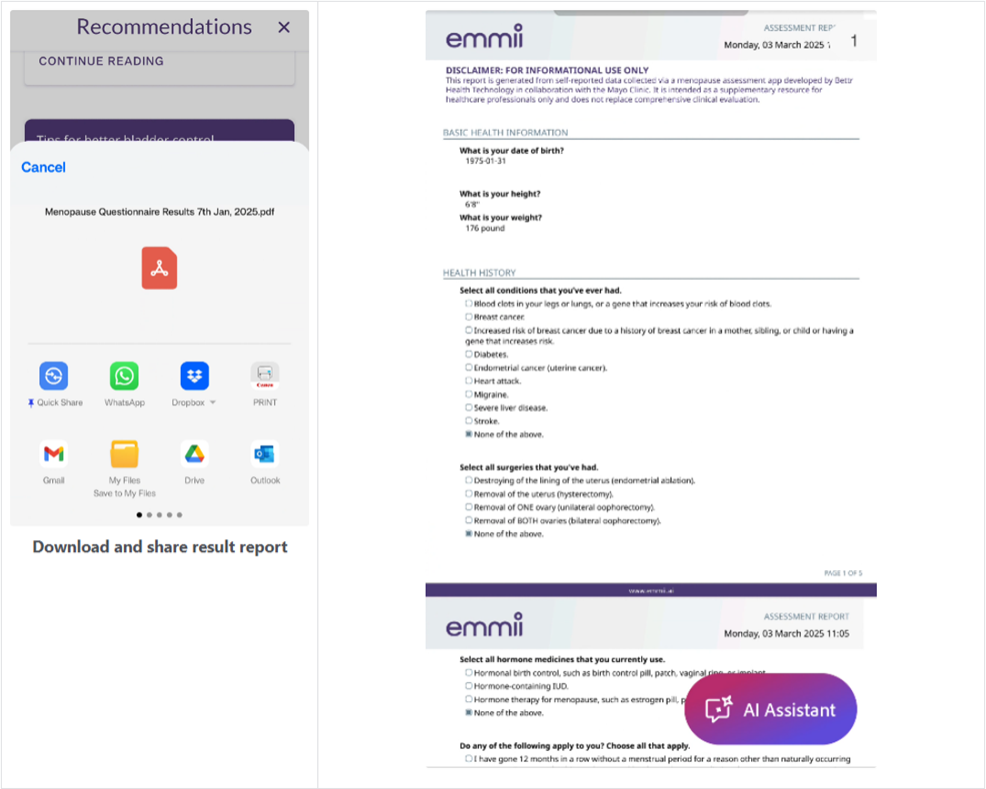
Screenshot of the discussion guide generated based on user input in the emmii app.

The app includes several key features designed to guide patients through their menopause journey. Features were selected based on their potential to educate users and improve clinical communication about bothersome menopause symptoms. Users initially enter basic health information, including height, weight, personal medical history, and current use of menopause treatments (Figure 1). Then, they rate their menopause-related symptoms by using the Menopause Rating Scale (MRS) [27] (Figure 2). Based on user input, the app generates personalized, evidence-based recommendations that include lifestyle modifications, hormone therapy, and nonhormone treatment options (Figure 3). A distinctive feature of emmii is its ability to also generate a personalized discussion guide (Figure 4), which users can share with their primary care clinicians to facilitate meaningful conversations about symptom management and treatment preferences.

### Beta Testing

The app underwent beta testing using Apple’s proprietary TestFlight software (Apple Inc) and was tested on Android devices to ensure cross-platform functionality. A convenience sample of 24 women aged 40 years and older was recruited via email. Beta testers were asked to focus on app usability and therefore not required to meet a specific MRS threshold or have an upcoming primary care appointment. Individuals were recruited via email, and those who agreed to participate were provided with detailed instructions for downloading and accessing the app using a unique code. No incentives were offered for participation. After using the app, all beta testers completed a survey that included the System Usability Scale, a validated 10-question survey tool commonly used to assess perceived utility, design, and overall experience [28]. System Usability Scale scores above 50 are generally considered acceptable. The average System Usability Scale score among patients was 59, with a range from 35 to 88, indicating generally acceptable usability. A subset of 10 participants who completed the postappointment survey agreed to participate in follow-up interviews during which they provided more details about their experience with the app. These interviews lasted an average of 10 minutes and were conducted between 1 and 3 weeks following their engagement with the app.

The primary purpose of the beta testing period was to iteratively improve the interface based on feedback from beta testers, and several significant modifications were made to the app during this period. Key changes included improving data entry fields to better accommodate the input of height and weight, resolving display issues with the “Get My Results” button, improving onboarding with a new welcome page, adjusting font settings so that users could enlarge the fonts without sacrificing functionality, and modifying entry fields so that users could save and edit their information. These improvements were aimed at enhancing the app’s accessibility and aligning it with the needs and expectations of midlife users.

### Study Design and Participants

This prospective randomized trial will identify women aged 45-55 years with primary care appointments scheduled within 3 weeks of enrollment at one of the several geographic locations of Mayo Clinic. Eligible patients will be identified through the electronic health record (EHR) system and contacted via email with a link to complete the MRS and provide informed consent. Women with a total MRS score ≥5, indicating bothersome menopause symptoms, will be randomized to one of the 2 groups: the intervention group (n=200), which will receive access to the emmii app, or the control group (n=200), which will receive an educational pamphlet. The pamphlet includes educational content on perimenopause and menopause symptoms, hormone and nonhormone treatment options, and lifestyle strategies, based on clinical guidelines. Although it offers similar content to the app, it does not include emmii’s interactive or personalized features. A copy of the full pamphlet is provided in Multimedia Appendix 1, and the SPIRIT (Standard Protocol Items: Recommendations for Interventional Trials) checklist is provided in Multimedia Appendix 2 for reference.

This is an unblinded trial; patients will be notified of their group assignment immediately after randomization. A formal sample size calculation was not conducted due to the absence of pilot data and the exploratory nature of the trial, which includes a range of primary and secondary outcomes of interest. The target sample size of 200 patients per group was based on anticipated recruitment feasibility within the study timeline.

### Inclusion and Exclusion Criteria

Patients must be English-speaking, aged 45-55 years, have a qualifying primary care appointment, meet the MRS symptom threshold (≥5), and provide informed consent. Exclusion criteria include inability to provide consent or unwillingness to engage with the app.

### Recruitment

Eligible patients will receive an email invitation with a secure link to complete the screening MRS and provide consent. Follow-up phone calls may be made to ensure that enrollment targets are met. Recruitment will continue on a rolling basis until the target sample size is reached.

### Study Procedures

Patients randomized to the intervention group will be emailed instructions on how to download and use emmii. The app will guide users through symptom reporting and generate a personalized menopause discussion guide, which patients will be instructed to bring to their upcoming primary care appointments. Patients in the control group will be emailed a digital copy of the menopause education pamphlet.

Patients in both intervention and control groups will be asked to complete a postappointment survey within 1-3 weeks of the appointment date to identify whether they used the menopause discussion guide (from the app) or pamphlet. The surveys will also assess whether menopause symptoms were discussed during the visit, their priorities for menopause-related health care, their perceived access to menopause care, and preferences for receiving information on menopause symptoms and treatment options. App usage metrics, including frequency of use and features accessed, will also be collected for patients in the intervention group to explore potential associations between engagement and outcomes. Patients will receive up to 3 reminder messages and will have up to 3 weeks after their appointments to complete the survey.

After the appointment, the primary care clinician who provided care to the patient will also be asked to complete a brief postappointment survey to assess whether the app-generated guide or pamphlet was used during the encounter and evaluate their confidence in managing menopause-related symptoms in both scenarios.

### Outcome Measures

Outcomes will be assessed from 3 main sources: patient surveys, clinician surveys, and EHR data. In the intervention group, adherence will be captured by BettrHealth, which will share information on whether individuals in the treatment group accessed the app and completed it to the point of receiving personalized recommendations. Adherence will be further assessed in more detail through patient-reported measures on the postappointment survey. App engagement metrics, including frequency of use and feature interaction, will be tracked through the app’s built-in analytics platform. Outcome measures, including data sources, purpose, and timeline for measurement and data collection, are presented in Table 1.

**Table 1:** Outcome measures, data sources, and timeline for emmii app evaluation.

Data source	Outcome measures	Purpose	Timeline
Menopause Rating Scale	Symptom severity across somatic, psychological, and urogenital domains; includes 11 items scored 0 (absent) to 4 (very severe); total score range: 0-44.	Determines eligibility (≥5 threshold) and customizes participant information sheets after app engagement.	Pre-enrollment (intervention and control groups) and after app use (intervention group)
Patient survey	Patient use of the app-generated menopause discussion guide versus pamphlet, menopause-related health care priorities, perceived access to care, and information preferences.	Evaluates patient engagement with intervention materials, preferences for menopause care and information, and satisfaction with care.	After primary care appointment (intervention and control groups)
Clinician survey	Encounter use of the app-generated menopause discussion guide versus pamphlet, confidence in managing menopause-related symptoms.	Evaluates clinician perception of the intervention materials, satisfaction with the shared decision-making tools, and perspective regarding menopause treatment.	After primary care appointment (intervention and control groups)
Electronic health record	Prescription rates for hormone therapy and nonhormonal treatments (ie, serotonin reuptake inhibitors/serotonin-norepinephrine reuptake inhibitors, gabapentin, oxybutynin, fezolinetant) for menopause symptoms, linked to survey data.	Assesses treatment patterns and correlates them with the use of the menopause app versus pamphlet alone.	Collected retrospectively 6 months after the appointment (intervention and control groups)

### MRS Questionnaire

MRS is a validated self-administered questionnaire used to assess the severity of menopause-related symptoms. Study participants will take this survey prior to enrollment to determine eligibility. MRS is composed of 11 distinct items across 3 symptom domains: somatic, psychological, and urogenital symptoms [27]. Each item is scored on a severity scale ranging from 0 (absent) to 4 (very severe), with increasing severity scores of 1 (mild), 2 (moderate), 3 (severe), and 4 (very severe). The overall score, which ranges from 0 to 44, is calculated by summing the individual item scores, with a higher total indicating a greater burden of menopause symptoms. For this study, a total MRS score of ≥5 will be used as the threshold to determine eligibility, indicating the presence of bothersome menopause symptoms warranting further evaluation. Patients will also take the survey again after accessing the app to enable the generation of personalized treatment recommendations tailored to their reported symptoms.

### Patient-Reported Outcomes

A survey will be administered after the primary care appointment to assess how patients interacted with the app-generated discussion guide compared to the pamphlet. It will also capture their treatment preferences and perceptions of access to menopause care. This outcome will evaluate the effectiveness of the intervention in increasing patient engagement and shared decision-making.

### Clinician-Reported Outcomes

A survey will be administered after the primary care appointment to assess how clinicians interacted with the app-generated discussion guide compared to the pamphlet. It will also inquire about the general confidence in treating menopause symptoms. This outcome will assess the clinician’s perception of the intervention and satisfaction with the shared decision-making tool.

### Clinical Outcomes (EHR-Based)

Clinical outcomes will be assessed by linking survey responses to EHR data to assess treatment outcomes that occur after appointments among those using the app and compare them to the control group. Specific outcomes include prescription rates of menopause-related pharmacologic treatments (hormone therapy and nonhormone treatments, including serotonin reuptake inhibitors/serotonin-norepinephrine reuptake inhibitors, gabapentin, oxybutynin, fezolinetant) among intervention and control groups. These data will allow the comparison of treatment patterns and explore whether app usage influences patient preferences and clinicians’ prescribing patterns.

### Data Collection

This study includes both existing data at the time of institutional review board submission and newly collected data. Existing appointment data will be used to determine eligibility, and demographic data will be incorporated into the analysis. Newly collected data will be gathered through participant surveys conducted after their appointments, as well as through the extraction of clinical outcome data from the EHR to evaluate treatment outcomes.

### Data Analysis

Descriptive statistics will be used to analyze cross-sectional survey data and summarize participant characteristics. Statistical tests, including chi-square tests and Wilcoxon rank sum tests, will be used to compare treatment outcomes between intervention and control groups, where groups will be based on randomization (ie, intention to treat). Multivariable models will be applied to assess differences in the outcomes after adjusting for baseline differences in MRS scores and demographics (including age and self-reported race/ethnicity) between groups. Missing survey responses will not be imputed. All data analyses will be performed using SAS software (version 9.4; SAS Institute, Inc).

### Ethical Considerations

This study was approved by the Mayo Clinic Institutional Review Board (approval ID: 24-013662). Informed consent will be obtained from all study participants. The consent process involves 2 main components: (1) the initial documentation of informed consent through Remote Electronic Consent technology, ensuring participant comprehension and agreement; and (2) for those who elected to participate, an oral consent script will be used to reinforce key study details, followed by the collection of a signed Health Insurance Portability and Accountability Act authorization. To protect participant privacy, all collected data will be deidentified prior to analysis and securely maintained on an encrypted server. No compensation will be offered to study participants. No identification of individual participants is included in the images, figures, or supplemental materials of this manuscript. All potential participants will receive explicit information regarding the voluntary nature of their involvement. It will be clearly communicated that participants have the right to withdraw their consent or cease participation at any point during the study, without incurring any penalty.

## Results

The beta testing for emmii content and functionality was conducted from October through December 2024. Feedback collected during this phase led to several updates to the app, which were implemented in January and February 2025. Data collection for the randomized controlled trial is scheduled to begin in April 2025.

## Discussion

### Overview

By integrating symptom tracking, evidence-based educational content, and a personalized discussion guide, the emmii app has the potential to enhance patients’ knowledge about treatment options for menopause symptoms. This may result in shared decision-making with their primary care clinicians regarding treatment options, including both hormonal and nonhormonal therapies. Many mHealth apps lack evidence-based content or medical expertise; however, emmii was developed by physicians at Mayo Clinic who are certified by The Menopause Society, ensuring that the app provides evidence-based, clinically relevant information. Given that many women may hesitate to discuss menopause symptoms in a health care setting, emmii offers a valuable tool for facilitating structured and informed conversations between patients and their primary care clinicians [29].

Although the strengths of the emmii app are clearly defined, evaluating its impact will be essential to understand its role in improving patient outcomes such as symptom management and treatment adherence. These results will help determine the app’s broader applicability and its potential for widespread implementation in diverse health care settings. Because it is designed as a self-guided mHealth app, emmii has the potential to be scaled across various health care systems with minimal additional resource demands. Future integration of the app into EHR portals or patient intake workflows could optimize its use in primary care settings. Cost-effectiveness analyses will be essential to determine whether the app can reduce unnecessary clinical visits or improve adherence to effective treatments. Based on the study’s findings, future adaptations may include culturally tailored content, language translation, or integration with telehealth platforms to further expand reach and clinical utility.

### Limitations

Although this study will present an innovative approach by integrating evidence-based content, symptom tracking, and personalized discussion guides, there are several potential limitations. Despite its potential, successful implementation of the emmii app must consider barriers related to digital access and literacy. Disparities in smartphone ownership, internet access, and comfort with using technology may disproportionately affect older adults, those from lower socioeconomic backgrounds, and individuals from underrepresented racial and ethnic groups. These challenges could limit engagement and create inequities in app-based menopause care. To address these barriers, future efforts should explore providing digital literacy support, offering the app in multiple languages, and ensuring that accessibility features are embedded into the platform. Partnering with community health centers and integrating the app into existing patient education workflows may also facilitate access for underserved populations. Reliance on self-reported symptom data may introduce bias, as patients’ engagement with the app may vary based on factors such as symptom severity, comfort with technology, and personal preferences. Additionally, the study’s focus on patients with upcoming primary care appointments within a specific health care system may limit the generalizability of the findings, as the sample may not fully represent the broader population of women experiencing menopause. Moreover, the potential lack of cultural and racial diversity among the patients could limit the applicability of the results to all women.

### Conclusion

This protocol paper outlines the use of emmii, an innovative mHealth app designed to improve the menopause experience for midlife women. The app offers a comprehensive approach to managing menopause symptoms by addressing knowledge gaps, tracking symptoms, and facilitating communication between patients and primary care clinicians. This tool has the potential to enhance clinical interactions, support shared decision-making, and ultimately improve patient experience and quality of life.

## Appendix

Multimedia Appendix 1 Educational menopause pamphlet provided to patients in the control group.

Multimedia Appendix 2 SPIRIT checklist.

## Abbreviations

EHR: electronic health record
mHealth: mobile health
MRS: Menopause Rating Scale
SPIRIT: Standard Protocol Items: Recommendations for Interventional Trials
