# Supplementary material for: Evaluating the Efficacy of a Mobile Phone App in Enhancing Menopause Knowledge and Shared Decision-Making: Protocol for a Randomized Controlled Trial
Source: JMIR Res Protoc. 2025 Oct 8;14:e76536. doi: 10.2196/76536 (PMC12547328; doi:10.2196/76536)
Supplement: Multimedia Appendix 1 [file resprot_v14i1e76536_app1.pdf]

MAYO CLINIC

PATIENT EDUCATION

Perimenopause and Menopause

EDUCATION
learning
EXCELLENCE
HEALTHY
CARING
INTERACTION
LIVING

BARBARA WOODWARD LIPS
PATIENT EDUCATION CENTER

Introduction

Menopause is a natural biological process for women. It is not a single event, but rather a series of changes that can start in your 30s or 40s and last into your 50s or 60s.

This information is intended to help you understand menopause, as well as give you information you can use to stay healthy and improve your sense of well-being during this important time in your life.

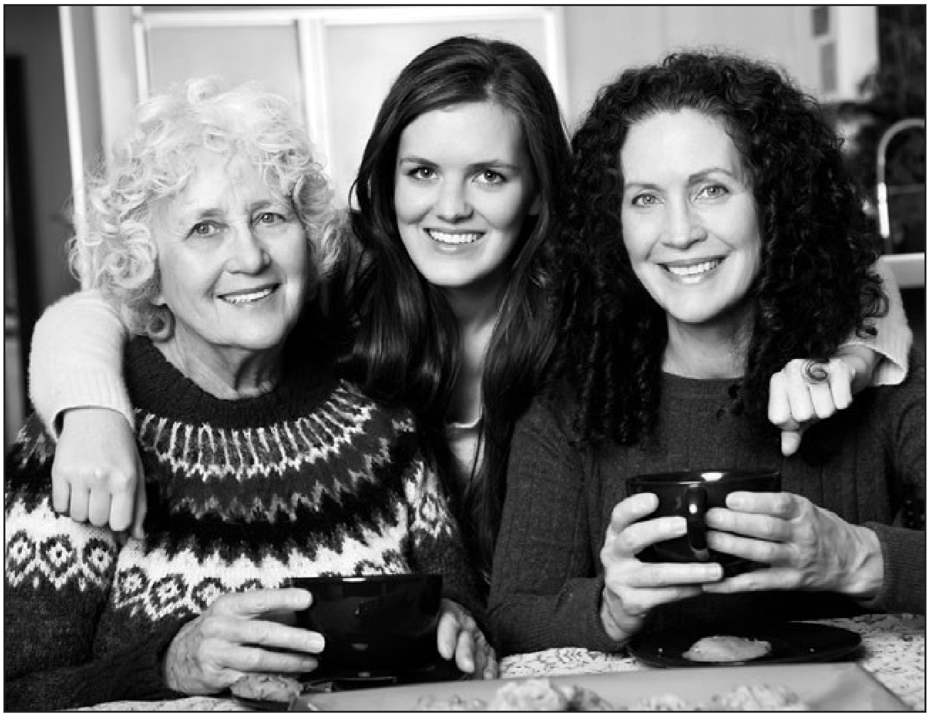

The Menstrual Cycle

The **ovaries** are two glands, one on each side of the uterus, that store eggs and make the hormones **estrogen** and **progesterone** from puberty until menopause. (Hormones are chemicals that carry messages throughout the body.) Changing levels of these hormones control the **menstrual cycle**, which includes **ovulation** (egg released from the ovary) and **menstruation** (menstrual period).

During a normal menstrual cycle, estrogen prepares the uterine lining for a possible pregnancy. When the estrogen level reaches a certain point, one ovary releases a ripened egg (ovulation). The level of progesterone starts to rise. If the egg is not fertilized, the progesterone level drops, causing the thickened uterine lining to shed (menstruation).

The Stages of Natural Menopause

Health care providers define menopause as the time after 12 months have passed since your last period. The months or years leading up to menopause are called **perimenopause**. Perimenopause means around (peri) the end of menstruation (menopause). Perimenopause can last months, a few years, or up to six years or more.

During perimenopause, the number of egg-producing follicles in your ovaries diminishes, so the ovaries begin to produce less estrogen and progesterone. Your hormone levels rise and fall unevenly. This can lead to changes in your menstrual cycle (irregular menstruation) (figure 1). For example, some months the level of estrogen may be too low to result in ovulation, or the progesterone level may not be high enough to cause a period. You could have one or more of the following:

- Periods that are closer together (for example, more often than every 28 days)
- Periods that are further apart
- Bleeding that is heavier or lighter than you have had previously
- Bleeding that lasts shorter or longer than you have had previously

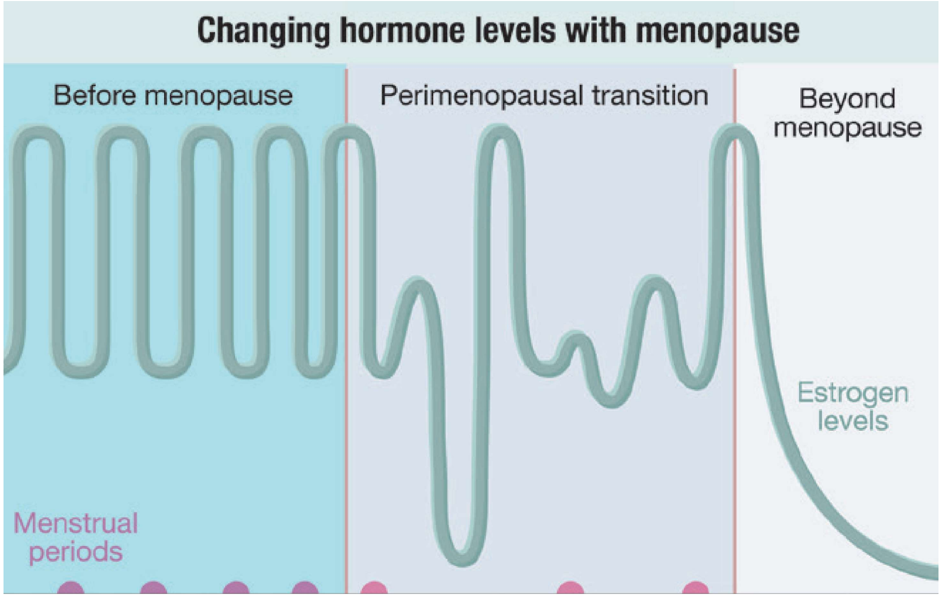

Irregular periods are often your first clue that menopause is approaching. Women taking birth control pills may have no change in their menstrual cycle.

You also may begin having other physical and emotional symptoms associated with a decrease of estrogen and progesterone. Women taking birth control pills may have milder symptoms or no symptoms at all. However, some women taking birth control pills have hot flashes during the hormone-free week of their cycle.

At some point, the ovaries stop making enough estrogen to thicken the lining of the uterus. This is when menstrual periods stop (figure 1). After 12 months have passed since your last period, you have reached menopause. Your ovaries produce much less estrogen and ovulation stops. Some estrogen continues to be produced by other parts of the body.

You may begin to notice signs of approaching menopause, such as hot flashes and menstrual irregularities, sometime in your 40s. But the start of declining estrogen levels may begin as early as your mid-30s. Most women experience natural menopause between ages 40 and 58 with the average age being about 51. Some women reach natural menopause as early as their 30s and some not until their 60s.

Induced Menopause

Certain surgical or medical treatments can bring on menopause earlier than expected. These include:

- **Total hysterectomy** — Surgical removal of the uterus, fallopian tubes and ovaries.
- **Bilateral oophorectomy** — Surgical removal of the ovaries.

Both surgical procedures cause menopause. There is no perimenopausal phase. Menstrual periods stop immediately, and a woman is likely to have menopausal symptoms, particularly hot flashes.

A hysterectomy that removes only the uterus, not the ovaries, does not directly cause menopause, because the ovaries continue to function and produce hormones even when menstrual bleeding no longer occurs. However, this type of hysterectomy may cause menopause to occur an average of two or three years earlier, probably by disturbing the blood supply to the ovaries. A woman who has had a hysterectomy does not have the most typical symptom of menopause — irregular periods — but may develop other physical symptoms, such as hot flashes.

- **Chemotherapy and pelvic radiation therapy**. These cancer therapies can damage the ovaries and result in an early menopause. Fertility and menstrual periods may end promptly during the course of cancer treatment or several months or years later.
- **Medications**. Some medications, such as antiestrogen medication, can cause permanent menopause. Other medications can cause temporary menopause while the medication is taken. An example is gonadotropin agonist, which is sometimes used to treat endometriosis or fibroids.

Premature Menopause

Menopause, whether natural or induced, is called premature when it occurs before age 40. Premature menopause can result from surgery, genetics, medical interventions or autoimmune conditions. Women who experience premature menopause spend more years without the protective benefits of estrogen and are at greater risk for certain health problems later in life.

Women who experience induced menopause or premature menopause are faced with menopause and its effects without time for gradual adjustment. Induced menopause causes abrupt loss of ovarian function, which tends to cause more intense symptoms. Premature menopause may not cause an abrupt onset of symptoms, but it can have a significant emotional impact and result in psychological distress.

Medical Care

Usually you don't need a laboratory test to confirm menopause. Menopause is confirmed when you have had no periods for one year. Perimenopause is suspected when periods become irregular and hot flashes start to occur.

Irregular periods are a hallmark of perimenopause. However, see your health care provider if:

- Bleeding is continuous and extremely heavy, for example, you're changing tampons or pads every hour.
- Bleeding lasts longer than 10 days.
- Bleeding occurs between periods.
- You have skipped periods but aren't sure you're in menopause.
- You have vaginal bleeding after you are menopausal.

Your health care provider may take a medical history, do a pelvic examination and possibly recommend a pregnancy test.

Rarely is blood testing needed or helpful to determine natural menopause. When blood testing to evaluate hormone levels around the time of menopause is needed, follicle-stimulating hormone (FSH) and estrogen (estradiol) levels may be measured. Levels of these hormones tend to be highly variable during perimenopause but once menopause occurs, FSH levels are increased, and estradiol levels are low.

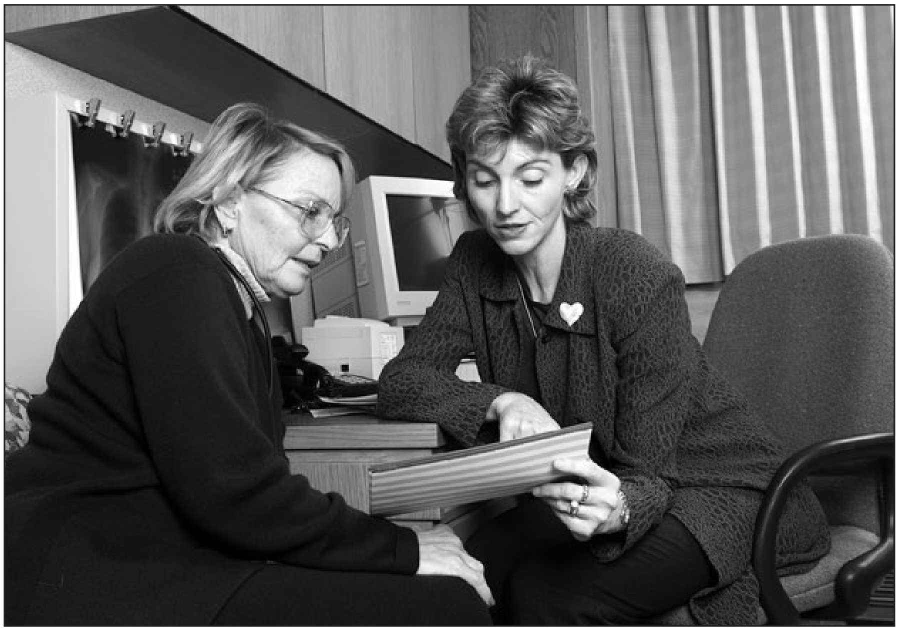

It may be helpful to chart your symptoms — menstrual periods, premenstrual symptoms, hot flashes, night sweats, sleep problems, vaginal dryness, mood changes, etc. The more attuned you are to your menstrual cycle and to symptoms, the less unpredictable perimenopause will seem.

Continue to see your health care provider during perimenopause and when you are in menopause for preventive health care, including regular pelvic and breast examinations. Although some problems attributed to aging are unavoidable, others can benefit from lifestyle changes and preventive therapies.

Symptoms of Perimenopause

Each woman experiences perimenopause and menopause differently. Some women have no symptoms. Others have one or more of the following physical and emotional changes.

- **Hot flashes** (also called hot flushes). Hot flashes are the most common symptom of menopause. Hot flashes are a feeling of warmth that moves upward from your chest to your shoulders, neck and head. You may sweat, and as the sweat evaporates from your skin, you may feel chilled, weak and sometimes slightly faint. Your face might look flushed, and red blotches may appear on your chest, neck and arms. The intensity, duration and frequency of hot flashes vary from person to person. Most hot flashes last from 30 seconds to several minutes, although they can last longer. You may have them a few times a month or several times a day. They can occur anytime, but usually are worse at night.
- **Sleep disturbances**. Many women experience disrupted sleep during perimenopause and after menopause. Sometimes it may be due to night sweats, hot flashes that occur during the night. You may awaken from a sound sleep with night sweats followed by feeling chilled. You may have difficulty falling back to sleep or achieving a deep, restful sleep. Some women going through menopause experience disrupted sleep even when not having night sweats. In either case, nonrestful sleep or lack of sleep can lead to irritability, mood changes and more trouble coping with menopausal symptoms during the day.

- **Vaginal and lower urinary tract changes**. As your estrogen level declines, the tissues lining your vagina and urethra — the opening to your bladder — become drier and thinner. With decreased lubrication you may experience burning, along with increased risk of infections of your urinary tract or vagina. Sexual intercourse may be uncomfortable or even painful. You may develop urinary frequency and urgency or sometimes incontinence.
- **Body and skin changes**. Aging causes decreased muscle mass, increased body fat, and the waist and abdomen to thicken. Lower estrogen level also affects collagen in your skin, so skin gradually becomes thinner and less elastic. You may notice a loss of fullness in your breasts, thinning hair and wrinkles in your skin. If you previously experienced adult acne, it may become worse. Although your estrogen levels drop, your body continues to produce small amounts of testosterone. As a result, you may develop coarse hair on your chin, upper lip, chest and abdomen.

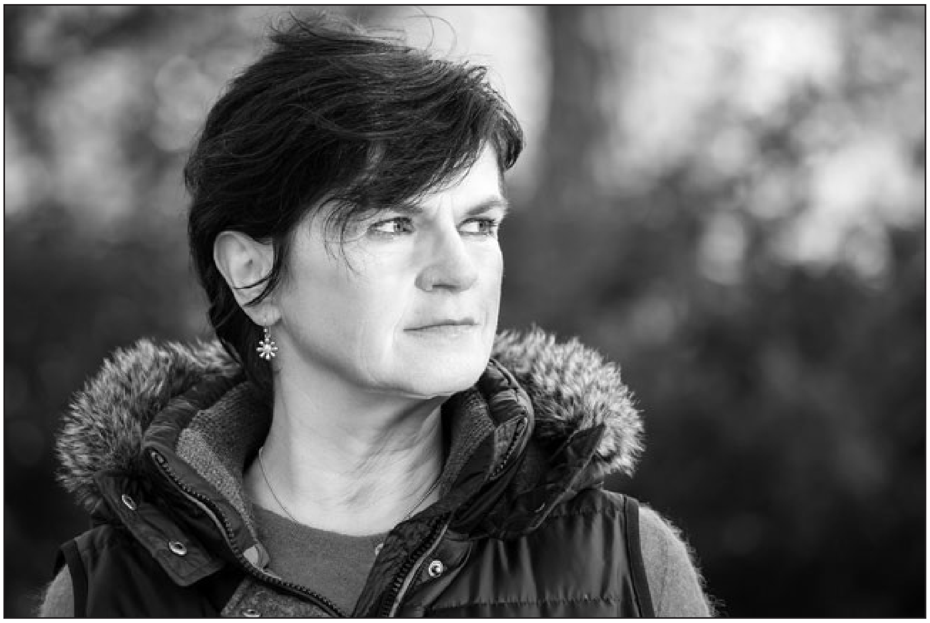

- **Emotional changes**. Some women experience mood swings or fatigue while experiencing menopausal changes, but emotional symptoms may arise during this time due to many other life factors than menopause itself. Stress, insomnia and life events that tend to occur in this stage of adulthood, such as the illness or death of a parent, grown children leaving home and retirement, all may contribute. For women with a past history of depression or mood disorders associated with hormonal changes, menopause may be an emotionally vulnerable time.

- **Changes in libido**. Sexual desire and arousal tend to decline with age in both men and women. For some women, interest in sex decreases with menopause, and for others, it increases. Most women who have enjoyed sex earlier in life continue to do so in menopause. There are many factors that can contribute to changes in sexual functioning at midlife and beyond. While some of the factors are hormonal, other important contributors to decreased interest in sex include availability of a partner, partner's changes in sexual functioning, medical conditions in either the woman or her partner, family or relationship stressors and many other life issues. Stress can reduce sexual desire at any time.
- **Decreased fertility**. As ovulation becomes less regular, your ability to become pregnant decreases. However, until you have gone a year without menstrual periods, you can potentially become pregnant.
- **Loss of bone density**. As your estrogen level declines, you may lose bone density, increasing your risk for osteoporosis.

Because subtle symptoms may come on gradually, you may not realize at first that they are interconnected with declining and fluctuating levels of estrogen and progesterone. You can do many things to make the most of this time of your life.

Relieving Symptoms and Caring for Yourself

Although menopause is associated with hormonal, physical and psychosocial changes in your life, menopause isn't the end of your life or your sexuality. You may spend as much as a third or more of your life after menopause.

More is known about menopause now than was known when your mother and grandmother experienced it. You can do more to relieve symptoms, avoid complications and improve your health and vigor during this important phase of your life.

Menopause itself requires no medical treatment. Instead, the focus is on relieving your symptoms, if necessary, and preventing or lessening chronic conditions that may occur during the menopausal years. For some women, the symptoms of perimenopause subside after entering menopause. In the meantime, try some of the following tips to help relieve symptoms. Your health care provider may suggest additional ways to ease symptoms.

- **Hot flashes**
  - During the day, wear lighter clothing or dress in layers that you can remove. Wear clothes made with natural fibers, such as cotton and linen. More clothing is being made with perspiration-wicking material. These fabrics may help you feel cooler.
  - Identify and avoid personal hot flash triggers (warm rooms, using a hair dryer, hot drinks, hot or spicy foods, alcohol, caffeine and cigarette smoking).
  - Try paced breathing (deep slow abdominal breathing) when a hot flash is starting.
  - Talk to your health care provider if these suggestions do not provide sufficient relief for hot flashes. Discuss the use of hormone therapy, certain antidepressant medications, and nonprescription vitamins and herbal therapies to relieve hot flashes. Using hormone therapy for a limited time is a reasonable option for most women who have significant menopausal symptoms.
- **Sleep disturbances**
  - Sleep in a cool room (keep a window open or use a ceiling fan). Keep a glass of water and small fan by your bed.
  - Try 100 percent cotton sleepwear (or a perspiration-wicking fabric), sheets and blankets for sleeping. Instead of one heavy cover, put a few thin blankets on the bed. Remove them as needed. Keep an extra set of bed clothes by your bed should you need to change during the night.
  - Talk with your health care provider if you have disturbed sleep unrelated to night sweats.

- **Vaginal changes**
  - Experiment with ways to increase your natural vaginal secretions before sexual intercourse.
  - Use a water-based lubricant, such as K-Y™ and Astroglide™, to help lubricate the tissues while having sexual intercourse.
  - Use vaginal moisturizers, such as Replens™ and Lubrin™, to help moisturize the vagina.
  - Talk with your health care provider about vaginal estrogen therapy, which is the most effective treatment for atrophic vaginitis symptoms that occur with menopause such as vaginal dryness and pain with intercourse. Vaginal estrogen therapy comes in several forms such as cream, tablet or ring that are inserted into the vagina.
  - Talk with your health care provider if you have vaginal itching or abnormal discharge.
- **Bladder changes**
  - To help prevent bladder problems, exercise your pelvic floor muscles (the muscles supporting the vagina, urinary opening and anus). This exercise, commonly known as Kegel's exercise, involves contracting or squeezing and then releasing the pelvic floor muscles in order to strengthen them. Ask your health care provider for more information on how to perform this exercise.
  - Limit fluid intake a few hours before bedtime.
  - Urinate promptly after sexual intercourse to prevent urinary tract infections.
  - Contact your health care provider if you have persistent discomfort with urination or any other troublesome bladder symptoms.

- **Body and skin changes**
  - Eat healthy foods — vegetables, fruits, whole grains, lean dairy products and lean meats, fish and other proteins (legumes).
  - Incorporate cardiovascular exercise, strength training and flexibility training into your weekly schedule.
  - Keep your body hydrated and well rested.
  - Apply moisturizer to your skin after bathing.
  - Apply sunscreen before going outside.
  - Change what you can or want to about your body and learn to accept the rest.
- **Emotional changes**
  - Decide what's most important in your life and base decisions on your values.
  - Strive for a healthy lifestyle — get plenty of sleep and exercise and eat healthy foods. Research has shown that even short periods of light exercise are beneficial for improving mood.
  - Limit or eliminate caffeine from your diet.
  - Stop smoking and limit alcohol.
  - Reduce stress through exercise, meditation, yoga, biofeedback, journaling, positive visualization, massage, or by taking a leisurely bath.

- Use your time and energy wisely. Try to minimize overcommitting yourself at home, work, church and community. Remind yourself to save some time for you. A healthy balance between obligations of work or caring for others and taking care of yourself is important to your emotional well-being.
- Try new activities (take a class, start a hobby, volunteer, or share your skills through community activities).
- Incorporate humor, joy, gratitude and forgiveness into your life. Lift your spirit by doing things you enjoy.
- Find support. Share concerns with family members or friends. Ask them for help, and ask them to be patient with you. Talk with other women your age or join a support group.
- If you feel depressed, talk with your health care provider. Menopause alone does not cause depression. However, women who are at increased risk of depression may experience a depressed mood during menopause. If you think you have symptoms of depression, talk to your health care provider. Treatment for depression is very effective and can help you enjoy life again.

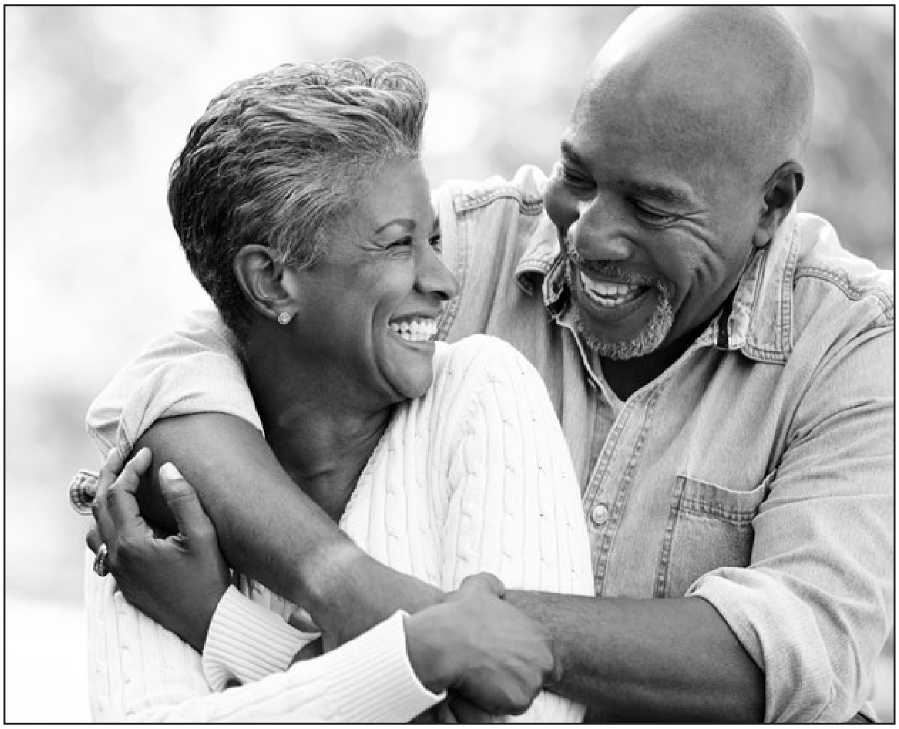

- **Changes in sexual desire**
  - Make time for your partner.
  - Talk with your partner about your relationship. Improved communication with your partner may help increase feelings of emotional closeness and intimacy.
  - Try incorporating more romance into your life with your partner.
  - Do the things that restore energy to your relationship — hold hands, listen to and help each other, go for walks together, dance together, speak words of appreciation, write a note, light candles and simply enjoy quiet time together.

- Reflect on your earlier relationship — the wonderful times and how you overcame the challenging times together.
- Experiment with ways to increase your sexual satisfaction and desire. This may include longer foreplay, use of lubricants, wearing lingerie, and relaxation techniques such as massage.
- If you have sexual concerns, talk with your health care provider and/or seek referral to a licensed sex therapist.

- **Decreased fertility**
  - If you are not menopausal, wish to become pregnant and are having difficulty conceiving, talk with your health care provider.
  - If you wish to avoid pregnancy, use a reliable form of birth control. Pregnancy is still possible until you haven't had a period for a year.
- **Bone density loss**
  - Before menopause, get at least 1,200 milligrams (mg) of calcium each day. After menopause, if you are not on hormone therapy, increase it to 1,500 mg. Take calcium supplements with a meal or snack.
  - An adequate amount of vitamin D (400-800 international units) is needed for good bone health. Sources of vitamin D include sunlight exposure, vitamin D-fortified milk, multivitamins, and some vitamin D-containing calcium supplements.

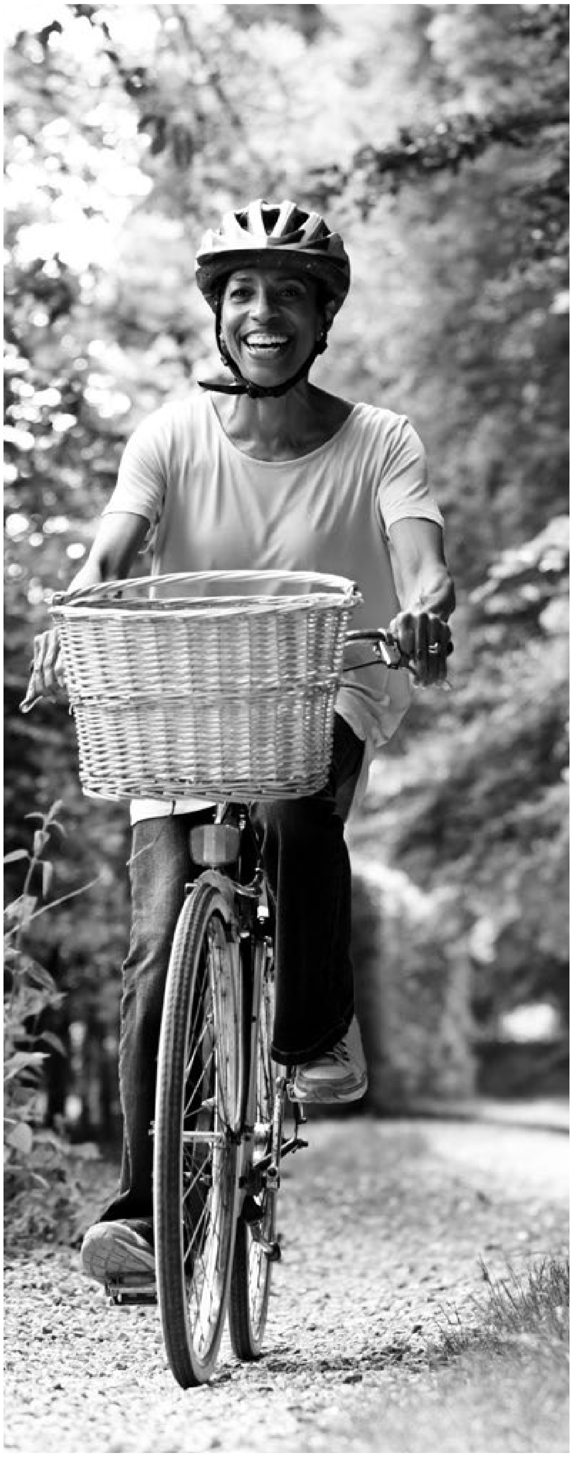

– Your calcium and vitamin D recommendations may be slightly different if you already have osteoporosis or osteopenia.

– Engage in regular weight-bearing exercise to keep your bones strong.

– Consider having a bone density test to check for osteoporosis or osteopenia (low bone density).

– Talk with your health care provider about bone-building medications and hormone therapy to help prevent bone loss.

– If you have low bone density or osteoporosis and are not already on treatment, talk with your health care provider about treatment options.

Health Conditions Associated With Menopause

Several chronic medical conditions tend to increase after menopause. By becoming aware of the following conditions, you can take steps to help reduce your risk.

- **Cardiovascular disease**. As your estrogen levels decline, your risk of cardiovascular disease increases. Coronary heart disease is the leading cause of death in women. Yet, you can do a great deal to reduce your risk of heart disease. These risk-reduction steps include stopping smoking, reducing high blood pressure, getting regular aerobic exercise, eating a heart-healthy diet and getting treatment for hyperlipidemia and diabetes. Ask your health care provider for a specific prevention and monitoring plan for you.
- **Osteoporosis.** During the first few years after menopause, you lose bone density at a much faster rate than earlier in life, which increases your risk of osteoporosis. Osteoporosis causes bones to become brittle and weak, leading to an increased risk of fractures. Postmenopausal women are especially susceptible to fractures of the hip, wrist and spine. That's why it is particularly important to get adequate calcium and vitamin D daily. It is also important to get regular weight-bearing exercise, such as walking, to keep your bones strong. Talk to your health care provider about having a bone density test to determine if you are at risk for osteoporosis.

- **Urinary incontinence.** As the tissues of your vagina and urethra become drier and thinner due to less estrogen, you may experience urinary incontinence. If your incontinence occurs mostly with coughing, laughing or lifting (stress urinary incontinence), Kegel exercises sometimes help by strengthening pelvic floor muscles. Ask your health care provider for more information on how to perform this exercise. Talk to your health care provider if you suspect prolapse problems (such as a sensation of bulging or pressure from the vagina).

  If your incontinence occurs mostly with a feeling of urgency or difficult getting to the bathroom in time, you may benefit from making changes in your fluid intake, frequency of urinating, or medications. Talk to your health care provider about other treatments that are available.

- **Weight gain**. As your body's metabolism — the rate at which you burn calories — slows and estrogen level declines, your body's weight and shape may change. You usually will need to eat fewer calories a day — as much as 200 to 400 fewer calories a day — and exercise more, just to maintain your current weight.

Conclusion

Perimenopause and menopause are natural transitions of your life. By educating yourself about menopause and what it means for your body and your life, you may be better equipped to deal with symptoms and life changes. This is an ideal time to begin or renew a health promotion program, take time for yourself, and do things that you have always wished you had time to do.

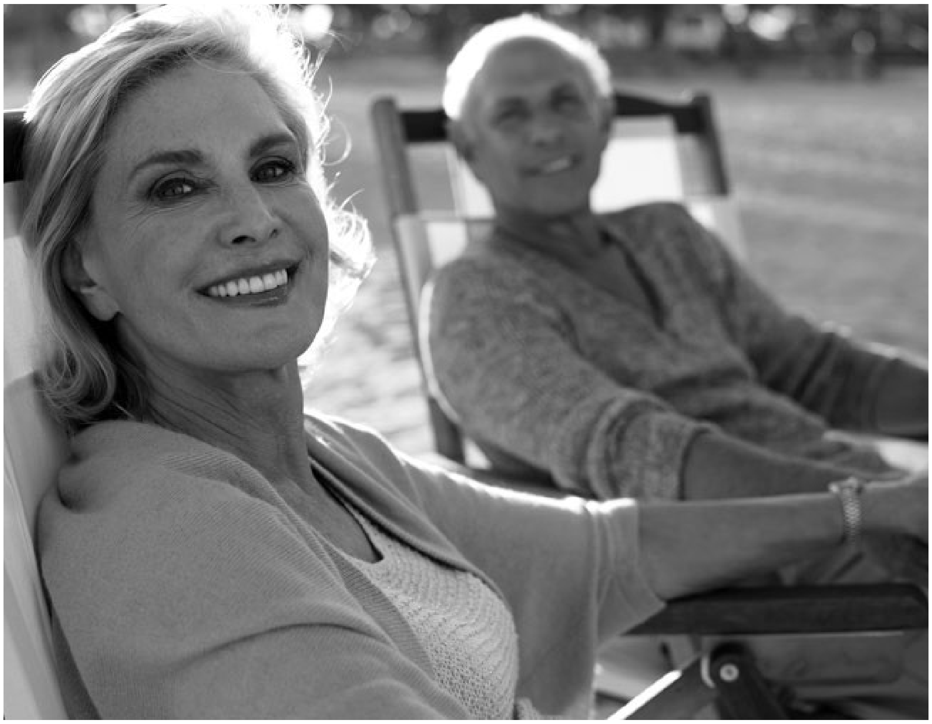

Notes

Notes

Notes

BARBARA WOODWARD LIPS PATIENT EDUCATION CENTER

Mrs. Lips, a resident of San Antonio, Texas, was a loyal Mayo Clinic patient of more than 40 years and a self-made business leader who significantly expanded her family's activities in oil, gas and ranching. Upon her death in 1995, Mrs. Lips paid the ultimate compliment by leaving her entire estate to Mayo Clinic. By naming the Barbara Woodward Lips Patient Education Center, Mayo honors her generosity, her love of learning, her belief in patient empowerment and her dedication to high-quality care.

This material is for your education and information only. This content does not replace medical advice, diagnosis or treatment. New medical research may change this information. If you have questions about a medical condition, always talk with your health care provider.

MAYO CLINIC | 200 First Street SW | Rochester, MN 55905 | mayoclinic.org

©2017 Mayo Foundation for Medical Education and Research. All rights reserved. MAYO, MAYO CLINIC and the triple-shield Mayo logo are trademarks and service marks of MFMER.

MC5544rev1017
